# Miniaturized NIRS Coupled with Machine Learning Algorithm for Noninvasively Quantifying Gluten Quality in Wheat Flour

**DOI:** 10.3390/foods14132393

**Published:** 2025-07-07

**Authors:** Yuling Wang, Chen Zhang, Xinhua Li, Longzhu Xing, Mengchao Lv, Hongju He, Leiqing Pan, Xingqi Ou

**Affiliations:** 1 School of Agriculture, Henan Institute of Science and Technology, Xinxiang 453003, China; wangyuling634@hist.edu.cn (Y.W.); lixinhua00530@163.com (X.L.); 2 School of Information Engineering, Xinxiang Institute of Engineering, Xinxiang 453700, China; zhangchen.hist@gmail.com; 3 School of Food Science, Henan Institute of Science and Technology, Xinxiang 453003, China; longzhu_xing@163.com (L.X.); mengchao_lv@163.com (M.L.); 4 College of Food Science and Technology, Nanjing Agricultural University, Nanjing 210095, China; pan_leiqing@njau.edu.cn

**Keywords:** wheat flour, NIRS, chemometrics, gluten, machine learning

## Abstract

This research implemented a miniaturized near-infrared spectroscopy (NIRS) system integrated with machine learning approaches for the quantitative evaluation of dry gluten content (DGC), wet gluten content (WGC), and the gluten index (GI) in wheat flour in a noninvasive manner. Five different algorithms were employed to mine the relationship between the full-range spectra (900–1700 nm) and three parameters, with support vector regression (SVR) demonstrating the best prediction performance for all gluten parameters (R_P_ = 0.9370–0.9430, RMSEP = 0.3450–0.4043%, and RPD = 3.1348–3.4998). Through a comparative evaluation of five wavelength selection techniques, 25–30 optimal wavelengths were identified, enabling the development of optimized SVR models. The improved whale optimization algorithm iWOA-based SVR (iWOA-SVR) model exhibited the strongest predictive capability among the five optimal wavelengths-based models, achieving comparable accuracy to the full-range spectra SVR for all gluten parameters (R_P_ = 0.9190–0.9385, RMSEP = 0.3927–0.5743%, and RPD = 3.0424–3.2509). The model’s robustness was confirmed through external validation and statistical analyses (*p* > 0.05 for F-test and *t*-test). The results highlight the effectiveness of micro-NIRS combined with iWOA-SVR for the nondestructive gluten quality assessment of wheat flour, providing a more valuable reference for expanding the use of NIRS technology and developing portable specialized NIRS equipment for industrial-level applications in the future.

## 1. Introduction

According to the Food and Agriculture Organization of the United Nations, the total global wheat production reached 0.8 billion metric tons in 2023, most of which is dedicated to milling applications [1,2]. As a major food processing raw material in the world, wheat flour is rich in carbohydrates, proteins, and dietary fibers and contains B vitamins (e.g., niacin, folic acid) and iron, zinc, selenium, and other minerals, which can not only quickly provide the energy needed by the human body but also promote digestion, metabolism, and immunity [3]. Whole-wheat flour, which retains the bran and germs, has a higher content of dietary fibers and micronutrients, further meeting the needs of a healthy diet [4]. As the cornerstone raw material of the food industry, wheat flour is processed into staple foods, snacks, and prepared foods through fermentation, baking, extrusion, and other processes, which supports a huge industrial chain and occupies an irreplaceable position in global food security and cultural inheritance [5].

Gluten is formed by the cross-linking of glutenin and gliadin in wheat flour after water absorption, and gluten content (GC) has been the basic standard for wheat flour classification (e.g., high GC, medium GC, low GC) [6]. Among these, dry gluten content (DGC) directly represents the absolute content of proteins in flour, while wet gluten content (WGC) reflects the water absorption capacity and network formation efficiency of gluten. The combination of DGC and WGC can reveal the “quality” and “quantity” of proteins in wheat flour [7]. GC also determines the processing adaptability of flour-based products. Specifically, a high GC gives dough strong malleability, which makes wheat flour more suitable for making bread, noodles, and other foods that need to be fermented or stretched. In contrast, low GC reduces dough toughness, making wheat flour more suitable for making cakes, biscuits, and other loose pastries [8].

The gluten index (GI) is the core index for evaluating the stability of the gluten network, and it reflects the quality stability of gluten by quantifying its elasticity and shear resistance [9]. A high GI indicates dense gluten structures and strong tensile resistance, while a low GI may cause the dough to collapse or the finished product to be loose in texture [10,11]. In addition, the GI is closely related to processing parameters [12]. High-GC flour with too low a GI requires the adjustment of mixing times or water temperatures to strengthen gluten cross-linking. Medium-GC flour with too high a GI needs the addition of starch or an emulsifier to soften the dough. The GI can also be used to predict the texture of final flour-based products [13]. For example, noodles made from high-GI flours are more chewable and resilient, while low-GI flours are more likely to achieve instant melting in the mouth. In summary, GC and the GI are not only the core indices for evaluating gluten quality but also the key parameters in the flour industry for optimizing processing technology, ensuring product consistency, and providing data support for the development of low-gluten or gluten-free alternatives.

The conventional methods available for determining the GC and GI of wheat flour mainly include manual water washing, drying and weighing, and centrifugal separation methods [14], which have significant drawbacks: complex operation, time consuming, significant human error, sample destructiveness, and insufficient accuracy. Although specialized instruments, such as gluten analyzers (Model GM 2200, Perten, Hägersten, Sweden), have been developed to measure gluten quality [15], they are oversized and lack portability and are often used in a laboratory, which makes gluten quality determination inapplicable when requiring industrial-scale and on-site detection. Compared with traditional methods, near-infrared spectroscopy technology (NIRS) has significant advantages and important value in detecting the quality of flour gluten. Characterized as fast and non-destructive, and without inflicting damage to the sample or chemical reagent, DGC, WGC, and GI can be determined simultaneously by mining NIRS data [16]. In addition, coupled with anti-interference machine algorithms such as support vector machine regression (SVR), random forest (RF), artificial neural network (ANN), lightweight gradient lift (LightGBM), and partial least squares (PLS) regression, NIRS has strong environmental adaptability, and it can ensure detection stability in complex scenarios such as fields and production lines. SVR is an extended application of support vector machines (SVMs) in dealing with regression problems and a typical implementation of the structured risk minimization idea in machine learning [17]. RF is an ensemble learning algorithm that improves the accuracy and robustness of the model by constructing multiple decision trees and integrating their prediction results. It can effectively avoid overfitting and has a strong tolerance for noisy data and missing values [18]. ANN is a machine learning algorithm that mimics the structure of biological neurons and can efficiently extract the deep correlations between spectral features and target components by simulating the parallel processing mechanism of the human brain [19]. LightGBM is an efficient gradient lift framework based on decision tree ensembles and has become an ideal tool for processing high-dimensional spectral data [20]. PLS regression is a statistical modeling method that integrates principal component analysis and multiple linear regression. PLS has become an irreplaceable classic method in NIRS analysis, providing an important reference baseline for subsequent nonlinear algorithms [21]. NIRS data typically contain hundreds to thousands of wavelength variables, among which a large number of adjacent wavelengths are highly correlated and carry repetitive information. Unfiltered full-wavelength modeling can lead to a sharp increase in model complexity, and redundant variables may mask effective signals, reducing the reliability of predictions. In addition, the full-wavelength model has difficulty meeting real-time demands due to its large amount of calculations. Therefore, wavelength selection is necessary to accelerate the prediction efficiency of the model by means of effective algorithms [22], such as principal component analysis (PCA), successive projection algorithms (SPAs), competitive adaptive reweighted sampling (CARS), recursive feature elimination (RFE), and improved whale optimization algorithms (iWOAs). PCA is a commonly used method for data dimensionality reduction and can be applied to screen the optimal wavelengths that contribute the most to spectral variation by analyzing the principal component loading in NIRS analysis [23]. SPA is a forward selection wavelength filtering algorithm, and a new wavelength with the lowest linear correlation with other selected wavelengths through the projection operation can be gradually selected to avoid information redundancy [24]. CARS is an intelligent wavelength screening method that combines Monte Carlo sampling and PLS regression. It identifies characteristic wavelengths by simulating the evolutionary mechanism of “survival of the fittest”, showing significant advantages in NIRS modeling [25]. RFE is a method for screening the optimal characteristic variables through iterative means and is often used for wavelength optimization in NIRS analysis [26]. iWOA is a swarm intelligence algorithm and is used for wavelength selection by simulating the predatory behavior of whales to optimize spectral feature selection [27]. While NIR calibration models inherently depend on training datasets comprising chemically analyzed reference samples to establish spectral property relationships, over-reliance on in-sample performance metrics introduces critical validation blind spots. Conventional model evaluation practices often fail to detect overfitting phenomena, necessitating rigorous external validation through completely independent datasets for unbiased performance quantification [28].

In recent years, the demand for food quality detection by portable miniaturized NIRS devices has been increasing [29]. By miniaturizing optical path design and embedding an artificial intelligence (AI) algorithm, miniaturized NIRS is designed and applied in multi-environment on-site detection, providing an immediate decision-making basis for wheat grading and flour quality control. To expand the application of NIRS in grain science, this study investigated the potential of machine learning-assisted portable miniaturized NIRS in tandem with wavelength reduction algorithms and independent validation for the rapid and efficient determination of the DGC, WGC, and GI of wheat flour. To our knowledge, this is the first attempt to apply miniaturized NIRS for the measurement of these key gluten quality indicators, which will further promote the intelligent and personalized development of wheat flour quality management.

## 2. Materials and Methods

### 2.1. Flour Preparation

Flour (80-mesh sieve; moisture, 12 ± 0.5%) of 10 different wheat varieties harvested from different places (Table 1) in the years 2023 and 2024 was provided by a local milling factory. Approximately 10 duplicate flour samples of each variety from each location and year were finally prepared, and a total of 1500 flour samples were obtained, with 500 used for each index evaluation.

### 2.2. NIRS Device Calibration and Spectral Acquisition

A portable miniaturized NIRS spectrometer (NIR-S-G1, InnoSpectra Technology, Hsinchu, Taiwan) was applied to acquire spectra (900–1700 nm). The device consists of several parts, mainly including a digital light processing-based post-dispersive optical engine, a Bluetooth low-energy module, a sampling module with a sapphire scan window, an illumination source (2 × 0.7 W, built-in tungsten filament lamps), a rechargeable lithium-ion battery pack, an operation panel, and an electromagnetic compatibility shielding case.

Prior to the experiment, the NIR device was connected via USB to a laptop running dedicated spectral acquisition software (ISC WinForms SDK GUI v3.9, InnoSpectra Technology, Taiwan) for system configuration. The spectrometer was calibrated using a standard white reference tile (99.99% reflectance) positioned in direct contact with its sapphire window. The instrument’s parameters were then optimized, with an exposure time of 0.635 ms and 10 averaged scans per measurement. For spectral acquisition, flour samples were uniformly layered on glass plates (diameter of 6 cm) and scanned five times through the spectrometer window, with the average spectrum recorded as the representative measurement. Through the systematic repetition of this protocol, 500 raw spectral datasets were obtained for each gluten quality indicator (DGC, WGC, and GI). Common preprocessing methods, such as Savitzky–Golay smoothing, standard normal variate (SNV), multiplicative scatter correction (MSC), normalization, and derivatives, were applied in a preliminary test, and no obvious improvements in model performance were found. As a result, all spectra were analyzed without preprocessing to preserve original signal features.

### 2.3. Measurement of DGC, WGC, and GI

After spectral acquisition, the DGC, WGC, and GI were determined using a Glutomatic 2200 system (Perten Instrument Ltd., Hägersten, Sweden). Each sample was measured three times and averaged for use.

### 2.4. Predictive Model Construction and Performance Evaluation

By inputting the data matrix comprising NIRS spectra and index values, five different algorithms, including SVR, RF, ANN, LightGBM, and PLS, were applied to mine the quantitative relationships between the NIRS spectra and the three indexes (DGC, WGC, and GI): That is, modeling was used. A novel algorithm named the artificial lemming algorithm (ALA) was applied to optimize the three parameters of SVR before SVR modeling. ALA is a meta-heuristic optimization algorithm inspired by the natural behaviors of lemmings. It seeks the optimal solution to complex problems by simulating the behaviors of lemmings, such as exploration, foraging, digging, and avoiding risks. It was used to automatically find the optimal hyperparameters in the SVR model to improve the predictive performance of the model [30]. The number of hidden layers/neurons was set as [50, 100, 200] for ANN. The key hyperparameters of LightGBM, including the number of trees, maximum depth of the tree, learning rate, number of leaf nodes, minimum sample number of leaf nodes, sample sampling ratio, and feature sampling ratio, were set as 200, 50, 0.1, 31, 20, 0.7, and 0.7, respectively. The predictive performances of these models were evaluated and compared in terms of correlation coefficients and root mean square errors in 5-fold cross-validation (R_CV_, RMSECV) and prediction (R_P_, RMSEP), as well as relative predictive deviation (RPD).

### 2.5. Optimal Wavelength Selection and Model Optimization

In this study, five different techniques, including PCA, SPA, CARS, RFE, and iWOA, were used to select optimal wavelengths from the full spectral range. After wavelength selection, the selected optimal wavelengths were input as predictor variables to optimize the original full-wavelength models. The predictive performances of these optimized models were evaluated and compared in terms of R^2^_CV,_ RMSECV, R^2^_P_, RMSEP, and RPD.

### 2.6. External Independent Validation

In this study, a dedicated validation set containing 50 flour samples for each index was randomly prepared from the same wheat varieties, locations, and years as the calibration/training set, prior to model development. The optimized model was subjected to external validation using these samples under the identical parameter configurations detailed in Section 2.4, ensuring methodological consistency while objectively assessing generalization capacity.

### 2.7. Two-Sample Test

In chemometric validation protocols, the F-test for variance homogeneity and Student’s *t*-test constitute a dual-test paradigm essential for comprehensive model diagnostics [30]. The F-statistic formally evaluates the null hypothesis (H0: σ^2^_predicted = σ^2^_reference) through the rigorous quantification of distributional variance equivalence, where the rejection of H0 signifies statistically significant variance heterogeneity (α = 0.05). Differently, the two-sample *t*-test assesses systematic prediction bias by comparing population means (μ_predicted vs. μ_reference), which is contingent upon fulfilling normality and independence assumptions. This integrated framework can systematically evaluate two pivotal reliability dimensions: variance stationarity (F-test) and central tendency accuracy (*t*-test). With collaborative applications of the F-test and *t*-test to provide diagnosis from different dimensions, the predictive precision and robustness of the optimized model were validated.

### 2.8. Statistical Analysis

The reference values of DGC, WGC, and GI were subjected to normal distribution and scatter diagram construction in software OriginPro 8.5 (OriginLab, Northampton, MA, USA). Spectral analysis and modeling were implemented via Scikit-learn’s feature_selection module within a Python 3.11.5 environment. The measured–predicted value pairs of the three indexes from simplified SVR models were statistically evaluated by a two-sample test protocol (F-test and *t*-test) in Microsoft Excel 2019.

## 3. Results and Discussions

### 3.1. Statistical Values of DGC, WGC, and GI

The measurement values of DGC, WGC, and GI were statistically analyzed, and 80% and 20% of the wheat flour samples were randomly classified by the scikit-learn tool and used for model training and prediction according to the preliminary experiments. The specific results are shown in Table 2.

**Figure 1:**
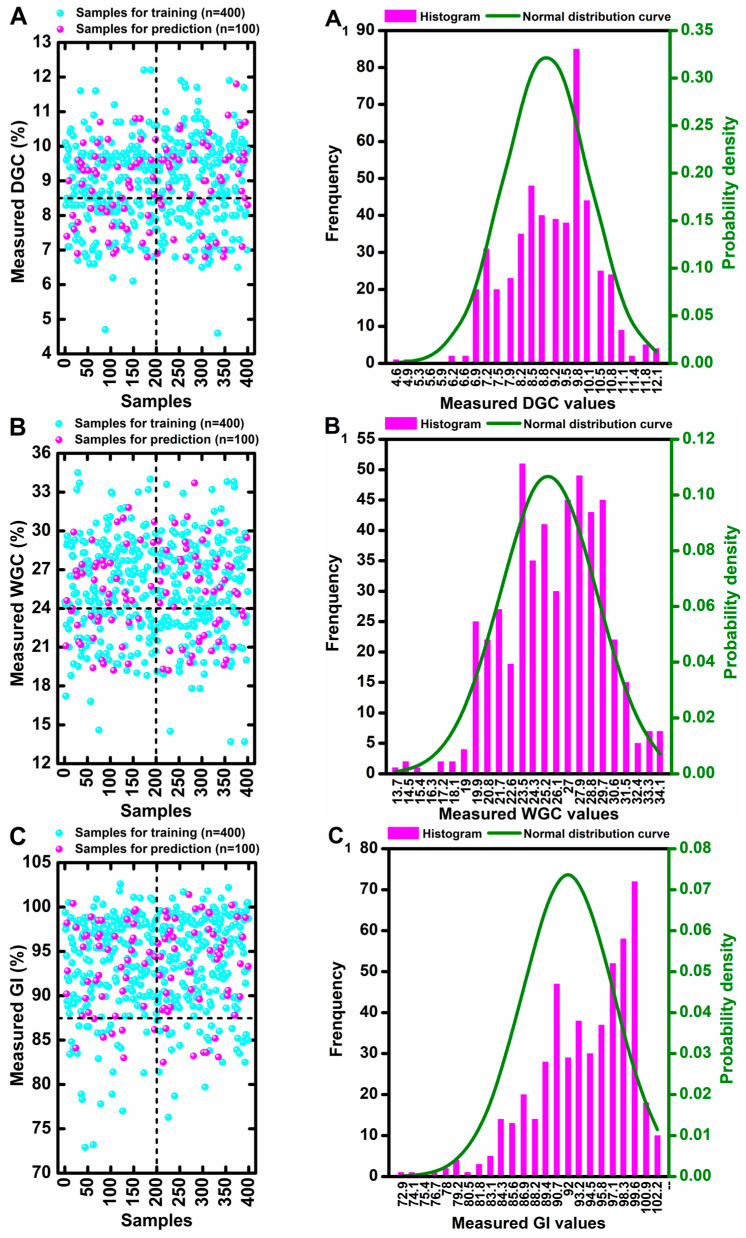
Scatters and normal distribution of wheat flour samples with different levels of DGC (**A**,**A_1_**), WGC (**B**,**B_1_**), and GI (**C**,**C_1_**).

The scatter distributions of these three parameters are shown in Figure 1A–C, with most of the values of DGC, WGC, and GI concentrated in ranges of 6.5–11%, 18–32%, and 80–102%, respectively. Furthermore, all measured values of these three indices followed a normal distribution, which are displayed in Figure 1A_1_–C_1_.

### 3.2. NIR Characteristics of Wheat Flour

**Figure 2:**
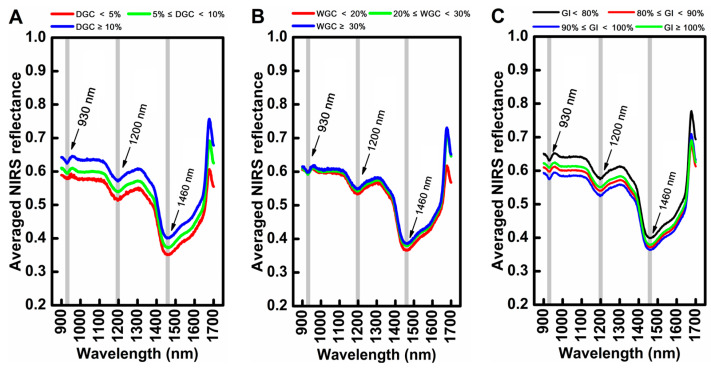
Averaged NIR spectral features of wheat flour samples with different levels of DGC (**A**), WGC (**B**), and GI (**C**).

The averaged NIR spectral characteristics of wheat flour samples with different levels of DGC, WGC, and GI in the 900–1700 nm range are exhibited in Figure 2. The curves of the spectral profiles showed similar trends of absorption for the three indexes, with three distinct bands centered at approximately 930 nm, 1200 nm, and 1460 nm. These absorption features primarily originated from the stretching vibrations of chemical bonds C-H, N-H, and O-H in the molecular structures of water and organic compounds. More specifically, the peaks presented at around 930 nm and 1200 nm resulted from a combination of O-H and C-H stretching. The peak appearing at about 1460 nm was assigned to O-H stretching [31].

Small positional variations among the spectral curves were also observed in these absorption peaks, which probably reflected the differences in chemical composition concentrations among the wheat flour samples. This spectral variability suggested potential for the quantitative analysis of component concentrations through appropriate chemometric methods.

Although no specific peaks that were directly related to the absorption of DGC, WGC, and GI were found within the 900–1700 nm range, the intrinsic relationship between each index and the NIRS spectra can be explored by applying chemometrics combined with machine learning algorithms based on the proportional relationship between protein content (reflecting N-H stretching) and gluten content in wheat flour [32].

### 3.3. Model Performance for Quantifying DGC, WGC, and GI Using Full Wavelengths

By executing five different modeling algorithms coupled with 5-fold cross-validation based on full wavelengths, the five models were established to perform prediction, and the detailed results are shown in Table 3.

It can be seen that the five models demonstrated different abilities in predicting each index, with SVR models performing the best for the three indices, exhibiting the highest values for R_P_ (0.9421 for DGC, 0.9436 for WGC, 0.9370 for GI) and RPD (3.2124 for DGC, 3.4998 for WGC, 3.1348 for GI), as well as the lowest values of RMSEP (0.3768 for DGC, 0.3450 for WGC, 0.4043 for GI). This indicated that the SVR algorithm performed better in mining the relationship between NIRS information and gluten quality parameters among the five algorithms. SVR was more suitable for predicting the DGC, WGC, and GI of wheat flour. Furthermore, it was found that there were both linear and nonlinear relationships between NIRS information and each gluten quality parameter. The quantitative relationship between them can be established by applying appropriate algorithms.

In recent studies, the spectral information of different ranges was investigated to predict the gluten quality of wheat flour, with a PLS modeling algorithm applied and good predictive results achieved, carrying R_P_ values of 0.90–0.95 for DGC [33,34] and 0.80–0.93 for WGC [35,36,37]. By comparison, parts of their results were similar or weaker than our study, which was probably caused by the use of different wavelengths and modeling algorithms. Although the DGC, WGC, and GI were simultaneously predicted by SVR using the spectra of 900–1700 nm in the latest study [38], however, the results were not as good as ours, and this may be due to the application of a different preprocessing method and the wheat variety. In addition, a miniaturized NIRS device was applied, and this was another obvious difference between our research and others. The purpose of our research was to explore the feasibility of the on-site application of miniaturized NIRS equipment in the gluten quality evaluation of wheat flour.

In summary, different research groups showed different results, all of which jointly promote the application of NIRS technology in the quality evaluation of gluten. Some factors, such as modeling algorithms and NIRS databases, are still challenges and should be constantly explored to accelerate NIRS applications.

### 3.4. Selection of Optimal Wavelengths by PCA, SPA, CARS, RFE, and iWOA

The optimal wavelengths were selected from the spectral data applied in SVR modeling by PCA, SPA, CARS, RFE, and iWOA, and the detailed results are shown in Table 4. Specifically, 25 optimal wavelengths were selected for DGC prediction, while 30 were selected for WGC and GI predictions. In other words, more than 92% of the wavelengths were reduced after wavelength selection, and only less than 8% of the wavelengths were retained for SVR model optimization for the three indexes.

**Figure 3:**
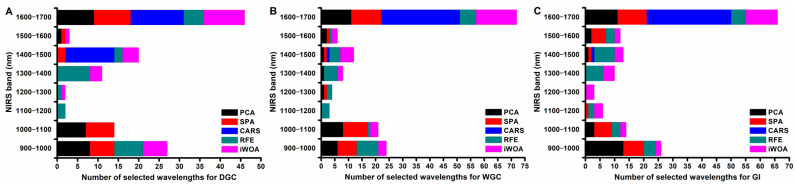
Distribution of optimal wavelengths selected by different methods for predicting DGC (**A**), WGC (**B**), and GI (**C**).

It can also be seen in Figure 3 that most of the optimal wavelengths selected by the five methods were mainly distributed in the three spectral regions of 900–1100 nm, 1300–1500 nm, and 1400–1700 nm for the three indexes. This phenomenon indicated that the three regions carried the most informative spectral data for the prediction of DGC, WGC, and GI. The spectral information within the three regions may contribute the most to the prediction. As reported, the 900–1100 nm range was related to the second overtone of N-H stretching vibrations, while the 1400–1700 nm range was related to the first overtone of the N-H stretching vibration in protein structures [39,40]. The combination vibration of N-H was found in the 1300–1500 nm range [41]. These works, combined with our study, demonstrated that the 900–1700 spectral range was indeed useful and can be mined through appropriate algorithms to achieve the rapid, nondestructive, and quantitative prediction of wheat flour gluten quality.

### 3.5. Model Performance for Quantifying DGC, WGC, and GI Using Optimal Wavelengths

Based on the selected optimal wavelengths, the original SVR models were optimized, and their performances are shown in Table 5. Among the five optimized SVR models, the SVR model built with optimal wavelengths selected by the iWOA method, that is, the iWOA-SVR model, exhibited the best capability in predicting each index, with R_P_ and RPD values being the largest and RMSEP being the lowest. By contrast, the other four optimized SVR models (PCA-SVR, SPA-SVR, CARS-SVR, and RFE-SVR) showed worse performances. This indicated that the iWOA method selected the most informative wavelengths from the full 900–1700 nm range compared with the other four methods (PCA, SPA, CARS, and RFE).

Compared with the SVR model for each index, the iWOA-SVR model showed slightly reduced accuracy and robustness (R_P_: 0.9385 vs. 0.9421 for DGC, 0.9357 vs. 0.9436 for WGC, 0.9190 vs. 0.9370 for GI; RMSEP: 0.5110 vs. 0.3768 for DGC, 0.3927 vs. 0.3450 for WGC, 0.5743 vs. 0.4043 for GI; RPD: 3.1159 vs. 3.2124 for DGC; 3.2509 vs. 3.4998 for WGC; 3.0424 vs. 3.1348 for GI), which meant that the optimal wavelengths selected by the iWOA method demonstrated similar performances in predicting the three indexes compared with the full-range wavelength. The reductions of more than 92% in wavelengths had a very small influence on the SVR model’s accuracy, further validating the importance and usefulness of wavelength selection in eliminating redundant data in NIRS analysis.

The results suggested that less than 8% of wavelengths can be used to replace the full-range wavelength in the modeling and prediction of the DGC, WGC, and GI of wheat flour. Furthermore, fewer wavelengths can lead to higher efficiency in modeling, and they are more suitable for developing rapid detection equipment.

### 3.6. Optimized SVR Models Validation Using Independent Samples

**Figure 4:**
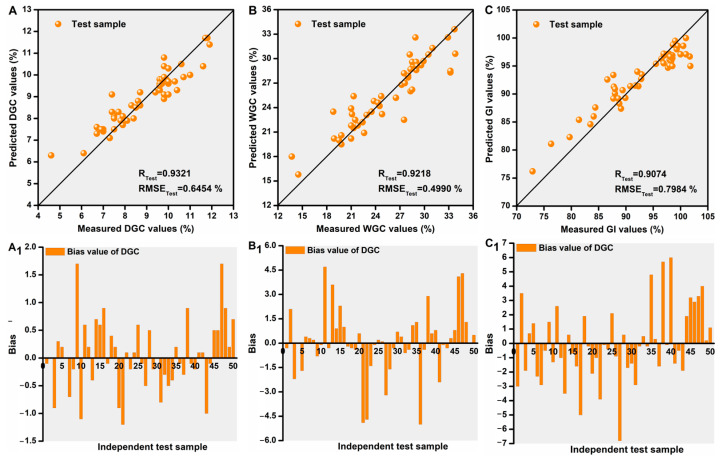
Accuracy and bias of optimized SVR models for predicting DGC (**A**,**A_1_**), WGC (**B**,**B_1_**), and GI (**C**,**C_1_**) based on independent samples.

A set of independent wheat flour samples (n = 50) per index was used to further validate the reliability and robustness of the three iWOA-SVR models. The validation performances of these models in predicting the three indices in terms of accuracy and predictive bias are shown in Figure 4. The values of DGC, WGC, and GI for the independent samples were well predicted (Figure 4A–C), with most of the bias values of the samples being less than 5% (Figure 4A_1_–C_1_).

### 3.7. Two-Sample Test Results

**Figure 5:**
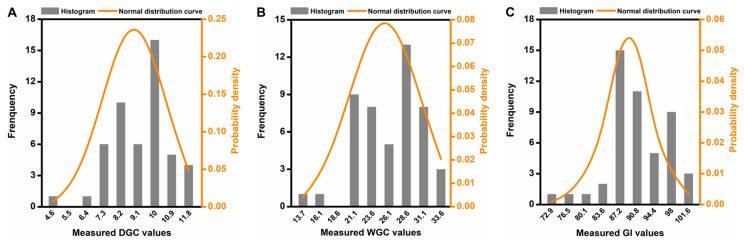
Normal distribution of independent wheat flour samples with different levels of DGC (**A**), WGC (**B**), and GI (**C**), respectively.

To further validate the ability of the three iWOA-SVR models, the F-test and *t*-test were performed, and the final results are shown in Table 6. It was observed that no statistically significant differences in variance between the measured and predicted values of the three gluten quality parameters were observed after the F-test, with the F value being less than the F ’one-tailed critical value’ (DGC: 1.5720 < 1.6073; WGC: 1.3711 < 1.6073; GI: 1.4213 ≤ 1.6073). Similarly, no statistically significant differences in the average between the measured and predicted values of the same indexes were observed after the *t*-test, with the *t* statistic below the *t* ‘two-tailed critical value’ (DGC: −0.1582 < 1.9845; WGC: −0.0888 < 1.9845; GI: 0.0336 < 1.9845). After statistical analysis, it was found that the 50 samples for each index basically followed a normal distribution (as shown in Figure 5), and this justified the reliability of the *t*-test results.

The two tests’ analysis results indicated the feasibility and reliability of the three iWOA-SVR models in quantifying the DGC, WGC, and GI of wheat flour.

## 4. Conclusions

This study developed a portable miniaturized NIRS system coupled with machine learning algorithms for nondestructively quantifying DGC, WGC, and GI in wheat flour. The relationship between the full wavelengths of 900–1700 nm and each index was mined by five algorithms, and the SVR model performed best in predicting each index, with the highest R_P_ and RPD and the lowest RMSEP. After wavelength selection by five different methods, 25–30 optimal wavelengths were selected, and the SVR models were optimized with fewer numbers of wavelengths. By comparison, the iWOA-SVR model showed the best performance among the five optimized SVR models, and it also has similar good accuracy in predicting each index compared with the original SVR model. The feasibility and reliability of the three iWOA-SVR models were further validated by a set of independent samples coupled with a two-sample test. This work demonstrated the great potential of the portable miniaturized NIRS combined with an SVR algorithm in predicting the gluten quality parameters of wheat flour.

Some challenges still exist in the rapid detection of flour quality by NIRS, and they need to be comprehensively improved from aspects of hardware performance, algorithm optimization, and standardization. (1) The spectral characteristics of different wheat varieties vary significantly, resulting in a decrease in the accuracy of the model during cross-variety detection, which can be improved by adopting military-grade materials in NIRS system design and optimizing the optical path to suppress stray light interference. (2) With respect to the spectra of multiple components in flour overlap, it is necessary to efficiently screen the key wavelength combinations to reduce redundancy and improve the signal-to-noise ratio. Existing models are still insufficiently adapted to sample diversity and need to be recalibrated when transferred to new wheat varieties or origins, which is time-consuming and costly. Combining transfer learning with deep learning to build cross-variety generalization models can reduce the need for recalibration. Developing multimodal fusion algorithms to effectively separate target component signals from background noise will be a research direction. (3) The lack of a unified calibration standard and the poor comparability of data among different NIRS devices have affected trust in industry applications. Integrating global wheat variety spectral data to promote the standardization of model generality is imperative and should be accelerated to drive the transformation from laboratory to industrial-level application.

## Figures and Tables

**Table 1:** Different wheat varieties harvested from different places.

Year	Wheat Variety	Location
2023 & 2024	Bainong 207 Bainong 307 Bainong 607 Bainong 697 Xinmai 26 Xinmai 45 Xinmai 58 Zhengmai 366 Zhoumai 36	Xinxiang City, Henan Province (113°56′ E, 35°18′ N)
	Bainong 627	Xinxiang City, Henan Province (113°56′ E, 35°18′ N) Anyang City, Henan Province (114°23′ E, 36°6′ N) Hebi City, Henan Province (114°18′ E, 35°44′ N) Luoyang City, Henan Province (112°27′ E, 34°37′ N) Xihua City, Henan Province (114°31′ E, 33°46′ N) Xuchang City, Henan Province (113°50′ E, 34°3′ N) Zhengzhou City, Henan Province (113°37′ E, 34°45′ N) Weinan City, Shaanxi Province (109°30′ E, 34°33′ N) Yangling City, Shaanxi Province (108°1′ E, 34°15′ N) Hefei City, Anhui Province (117°8′ E, 31°46′ N) Huainan City, Anhui Province (116°58′ E, 32°42′ N) Suzhou City, Jiangsu Province (120°35′ E, 31°19′ N) Huai′an City, Jiangsu Province (119°7′ E, 33°33′ N) Lianyungang City, Jiangsu Province (119°14′ E, 34°38′ N)

**Table 2:** Statistical values of measured DGC (%), WGC (%), and GI (%) for model training and prediction.

Index	Sample Set	Number of Sample	Range	Mean	Standard Deviation
DGC	Training	400	4.6–12.2	9.0	1.2
	Prediction	100	6.8–11.8	8.9	1.2
WGC	Training	400	13.7–34.5	25.6	3.8
	Prediction	100	19.2–33.7	25.3	3.5
GI	Training	400	72.9–102.6	93.5	5.4
	Prediction	100	82.5–101.4	93.2	5.0

**Table 3:** Predictive performance of machine learning models in predicting DGC (%), WGC (%), and GI (%) using the full wavelength.

Index	Number of Wavelengths	Model	Cross-Validation		Prediction		
			R_CV_	RMSECV	R_P_	RMSEP	RPD
DGC	360	SVR	0.9503	0.3276	0.9421	0.3768	3.2124
		RF	0.8468	0.5741	0.8174	0.7171	1.6880
		ANN	0.9156	0.4045	0.9087	0.4866	2.4877
		LightGBM	0.8556	0.6321	0.8528	0.5123	1.9150
		PLS	0.9377	0.3868	0.9172	0.4204	2.8792
WGC	360	SVR	0.9583	0.3853	0.9436	0.3450	3.4998
		RF	0.8159	0.6095	0.7815	0.6981	0.4874
		ANN	0.9163	0.4492	0.8864	0.4835	0.2337
		LightGBM	0.8921	0.5290	0.8402	0.5455	2.2135
		PLS	0.9436	0.4202	0.9006	0.3999	3.0192
GI	360	SVR	0.9387	0.4094	0.9370	0.4043	3.1348
		RF	0.7251	0.7116	0.6914	0.8077	1.4522
		ANN	0.8421	0.5317	0.8374	0.6325	1.8545
		LightGBM	0.7781	0.7354	0.7767	0.7368	1.5920
		PLS	0.8812	0.4784	0.8714	0.5545	2.1153

**Table 4:** Optimal wavelengths selected by PCA, SPA, CARS, RFE, and iWOA.

Index	Method	Optimal Wavelengths	Wavelength Reduction
DGC	PCA	929, 931, 954, 984, 987, 990, 993, 999, 1009, 1011, 1014, 1016, 1027, 1032, 1035, 1599, 1609, 1613, 1615, 1621, 1625, 1633, 1635, 1637, and 1641 nm	93%
	SPA	929, 956, 972, 977, 990, 993, 1009, 1011, 1014, 1021, 1032, 1056, 1099, 1474, 1495, 1588, 1613, 1619, 1621, 1623, 1625, 1633, 1641, 1655, and 1693 nm	93%
	CARS	1421, 1432, 1434, 1436, 1438, 1441, 1443, 1457, 1545, 1446, 1453, 1461, 1466, 1472, 1474, 1479, 1495, 1647, 1649, 1651, 1653, 1655, 1657, 1659, and 1660 nm	93%
	RFE	900, 905, 913, 926, 951, 977, 1046, 1119, 1123, 1227, 1306, 1325, 1340, 1344, 1347, 1358, 1368, 1370, 1407, 1497, 1625, 1647, 1651, 1655, and 1670 nm	93%
	iWOA	911, 916, 921, 924, 966, 1040, 1249, 1358, 1370, 1397, 1430, 1453, 1459, 1479, 1565, 1649, 1655, 1659, 1672, 1676, 1689, 1691, 1694, 1698, and 1700 nm	93%
WGC	PCA	929, 931, 934, 977, 990, 991, 1004, 1009, 1014, 1019, 1021, 1024, 1035, 1079, 1284, 1318, 1477, 1584, 1603, 1613, 1615, 1619, 1621, 1625, 1627, 1633, 1635, 1637, 1639, and 1641 nm	92%
	SPA	929, 966, 972, 977, 990, 993, 999, 1006, 1009, 1014, 1021, 1032, 1035, 1038, 1056, 1077, 1290, 1453, 1565, 1613, 1619, 1621, 1623, 1625, 1633, 1637, 1641, 1643, 1662, and 1689 nm	92%
	CARS	1446, 1647, 1649, 1651, 1653, 1655, 1657, 1659, 1660, 1662, 1664, 1666, 1668, 1670, 1672, 1674, 1676, 1678, 1680, 1682, 1683, 1685, 1687, 1689, 1691, 1693, 1694, 1696, 1698, and 1700 nm	92%
	RFE	905, 916, 921, 924, 929, 974, 991, 996, 1061, 1135, 1155, 1163, 1212, 1277, 1323, 1333, 1335, 1370, 1397, 1407, 1421, 1453, 1495, 1547, 1611, 1649, 1655, 1670, 1687, and 1698 nm	92%
	iWOA	929, 931, 951, 1001, 1040, 1073, 1306, 1368, 1402, 1405, 1443, 1446, 1459, 1581, 1594, 1635, 1655, 1657, 1666, 1668, 1670, 1674, 1676, 1680, 1575, 1689, 1691, 1694, 1696, and 1700 nm	92%
GI	PCA	921, 926, 929, 931, 937, 939, 942, 951, 966, 977, 991, 993, 999, 1006, 1021, 1038, 1402, 1581, 1590, 1605, 1609, 1611, 1613, 1621, 1623,1625, 1629, 1633, 1635, and 1649 nm	92%
	SPA	926, 929, 937, 939, 977, 991, 993, 1004, 1006, 1009, 1021, 1035, 1038, 1155, 1402, 1532, 1573, 1584, 1588, 1597, 1605, 1609, 1619, 1621, 1623, 1633, 1641, 1643, 1645, and 1676 nm	92%
	CARS	1414, 1645, 1647, 1651, 1653, 1655, 1657, 1659, 1660, 1662, 1664, 1666, 1668, 1670, 1672, 1674, 1676, 1678, 1680, 1682, 1683, 1685, 1687, 1689, 1691, 1693, 1694, 1696, 1698, and 1700 nm	92%
	RFE	911, 929, 944, 987, 1011, 1048, 1071, 1192, 1194, 1304, 1309, 1333, 1379, 1391, 1395, 1400, 1404, 1414, 1430, 1432, 1482, 1491, 1506, 1508, 1556, 1659, 1662, 1676, 1678, and 1685 nm	92%
	iWOA	931, 944, 1001, 1063, 1102, 1173, 1194, 1246, 1286, 1293, 1300, 1384, 1391, 1397, 1414, 1434, 1491, 1556, 1579, 1637, 1657, 1670, 1674, 1676, 1682, 1683, 1687, 1689, 1691, and 1696 nm	92%

**Table 5:** Predictive performance of machine learning models in predicting DGC (%), WGC (%), and GI (%) using the optimal wavelengths.

Index	Number of Wavelengths	Model	Cross-Validation		Prediction		
			R_CV_	RMSECV	R_P_	RMSEP	RPD
DGC	25	PCA-SVR	0.7551	0.8124	0.7592	0.8335	2.1522
		SPA-SVR	0.8893	0.6316	0.8815	0.6191	2.6552
		CARS-SVR	0.8186	0.5372	0.8730	0.7443	2.3263
		RFE-SVR	0.9288	0.4495	0.9184	0.5304	2.9820
		iWOA-SVR	0.9403	0.5096	0.9385	0.5110	3.1159
WGC	30	PCA-SVR	0.8016	0.7293	0.8011	0.7218	2.0728
		SPA-SVR	0.8634	0.5129	0.8353	0.6090	2.3825
		CARS-SVR	0.9027	0.431	0.8717	0.5196	2.7239
		RFE-SVR	0.9137	0.4084	0.9068	0.4905	2.8616
		iWOA-SVR	0.9399	0.2919	0.9357	0.3927	3.2509
GI	30	PCA-SVR	0.8521	0.6986	0.8614	0.7005	2.6745
		SPA-SVR	0.8742	0.5881	0.8697	0.6642	2.7659
		CARS-SVR	0.8775	0.7783	0.8808	0.6585	2.7813
		RFE-SVR	0.8883	0.5573	0.8868	0.6394	2.8344
		iWOA-SVR	0.9219	0.5251	0.9190	0.5743	3.0424

Note: PCA-SVR, SVR model based on optimal wavelengths selected by the PCA method; SPA-SVR, SVR model based on optimal wavelengths selected by the SPA method; CARS-SVR, SVR model based on optimal wavelengths selected by the CARS method; RFE-SVR, SVR model based on optimal wavelengths selected by the RFE method; iWOA-SVR, SVR model based on optimal wavelengths selected by the iWOA method.

**Table 6:** Two-sample test results for the measured and predicted values of the three indexes from the iWOA-SVR models.

Index	Model	Test	Item	Measured Value	Predicted Value
DGC	iWOA-SVR	F-test	Average	8.9	9.0
			Variance	2.6	1.6
			Observed value	50	50
			d*f*	49	49
			*F*	1.5720	
			*p* (*F* ≤ *f*) one-tailed	0.0584	
			F ‘one-tailed critical value’	1.6073	
		*t*-test	Average	8.9	9.0
			Variance	2.6	1.6
			Observed value	50	50
			Merger of variance	2.1	
			Assumed mean difference	0	
			d*f*	98	
			*t* Stat	−0.1582	
			*p* (T ≤ t) one-tailed	0.4373	
			*t* ‘one-tailed critical value’	1.6606	
			*p* (T ≤ t) two-tailed	0.8746	
			*t* ‘two-tailed critical value’	1.9845	
WGC	iWOA-SVR	F-test	Average	25.5	25.6
			Variance	23.7	17.3
			Observed value	50	50
			d*f*	49	49
			*F*	1.3711	
			*p* (*F* ≤ *f*) one-tailed	0.1364	
			F ‘one-tailed critical value’	1.6073	
		*t*-test	Average	25.5	25.6
			Variance	23.7	17.3
			Observed value	50	50
			Merger of variance	20.5	
			Assumed mean difference	0	
			d*f*	98	
			*t* Stat	−0.0888	
			*p* (T ≤ t) one-tailed	0.4647	
			*t* ‘one-tailed critical value’	1.6606	
			*p* (T ≤ t) two-tailed	0.9294	
			*t* ‘two-tailed critical value’	1.9845	
GI	iWOA-SVR	F-test	Average	93.0	93.0
			Variance	48.6	26.7
			Observed value	50	50
			d*f*	49	49
			*F*	1.4213	
			*p* (*F* ≤ *f*) one-tailed	0.0191	
			F ‘one-tailed critical value’	1.6073	
		*t*-test	Average	93.0	93.0
			Variance	48.6	26.7
			Observed value	50	50
			Merger of variance	37.6	
			Assumed mean difference	0	
			d*f*	98	
			*t* Stat	0.0336	
			*p* (T ≤ t) one-tailed	0.4866	
			*t* ‘one-tailed critical value’	1.6606	
			*p* (T ≤ t) two-tailed	0.9733	
			*t* ‘two-tailed critical value’	1.9845	

## Data Availability

The raw data that support the findings of this study are available from the corresponding author upon reasonable request.
